# Exploring the effects of geographic scale on spatial learning

**DOI:** 10.1186/s41235-020-00214-9

**Published:** 2020-04-05

**Authors:** Jiayan Zhao, Mark Simpson, Jan Oliver Wallgrün, Pejman Sajjadi, Alexander Klippel

**Affiliations:** Department of Geography, The Pennsylvania State University, Walker Building, 302 N Burrowes Street, University Park, PA 16802 USA

**Keywords:** Scale, Spatial learning, Virtual environments

## Abstract

**Background:**

Investigating the relationship between the human body and its spatial environment is a critical component in understanding the process of acquiring spatial knowledge. However, few empirical evaluations have looked at how the visual accessibility of an environment affects spatial learning. To address this gap, this paper focuses on geographic scale, defined as the spatial extent visually accessible from a single viewpoint. We present two experiments in which we manipulated geographic scale using two perspectives, a ground level and an elevated view, in order to better understand the scale effect on spatial learning. Learning outcomes were measured using estimates of direction and self-reports of mental workload.

**Results:**

In contrast to our hypothesis, we found few differences in spatial learning when comparing different perspectives. However, our analysis of pointing errors shows a significant interaction effect between the scale and spatial ability: The elevated perspective reduced the differences in pointing errors between low and high spatial ability participants in contrast to when participants learned the environment at ground level alone. Bimodal pointing distributions indicate that participants made systematic errors, for example, forgetting turns or segments. Modeling these errors revealed a unified alternative representation of the environment and further suggests that low spatial ability participants benefited more from the elevated perspective in terms of spatial learning compared to high spatial ability participants.

**Conclusions:**

We conclude that an increased geographic scale, which was accessible through an elevated perspective in this study, can help bridge the performance gap in spatial learning between low and high spatial ability participants.

## Significance

The research focuses on related but unstudied issues concerning the impact of visual accessibility of an environment (or geographic scale) on spatial learning. Compared to experiencing an environment at normal eye level (ground perspective), learning from an elevated perspective increases geographic scale given the larger spatial extent visually accessible from a single viewpoint. From an elevated perspective, a learner has access to a larger number of entities and their relations through direct observation, reducing the need to hold that information in memory. Offloading information into the environment has long been deemed critical for the efficient processing of information by the human cognitive system (Norman, 1993; Raubal & Worboys, 1999). However, this aspect of spatial learning in an environment space has yet to be examined. This study therefore experimentally examined the impact of ground vs. elevated perspective on spatial learning and systematically assessed how individual differences in spatial abilities modulated the scale effects. While our study did not show a significant positive effect of the elevated perspective, the interaction between perspective and spatial ability on learning performance has shown that the elevated perspective benefited learners with low spatial abilities more than those with high spatial abilities. We believe that the results will shed light on the design choices made within virtual navigation for bridging the performance gap between learners with different spatial abilities in various spatial or place-based learning tasks.

## Background

Scale is a key element in the acquisition of spatial knowledge (Bell, 2002). Geographers, psychologists, and other scientists interested in spatial learning define scale in multiple ways. Environmental data including maps, diagrams, and models rely on scale transformation in order to accurately represent spaces that are too large to be perceived from a single perspective (Bell, 2002). *Cartographic scale* is defined as the ratio between the distance on a map to the corresponding distance on the surface of the Earth (Lam & Quattrochi, 1992). Physical geographers (e.g., landscape ecologists) refer to scale as composed of two parts: *extent* and *grain* (Sayre, 2005). Extent refers to the relative size of a space or a phenomenon; grain refers to the finest level of spatial resolution available within a given data set. The former is also called *geographic scale*, which refers to the spatial extent of a phenomenon or a study. Using these definitions, a large-scale space would have a larger spatial extent than a small-scale space, with large extents generally implying a coarser grain due to the practical limits of sampling (Sayre, 2005).

People experience space in a scale-dependent way (Montello, 1993; Newcombe, 2018). In other words, people perceive relations between objects by relating the projective size of that environment to their body and action (e.g., looking, walking). Montello (1993) presents a classification of scale with respect to the projective size of the space relative to the human body: *figural spaces* (which are smaller than the body), *vista spaces* (which are visible from a single location), *environmental spaces* (which require movement to apprehend), and *geographical spaces* (which require an external representation like a map to understand).

Studies have demonstrated that human spatial abilities are not uniform across different scales (Hegarty, Montello, Richardson, Ishikawa, & Lovelace, 2006); for example, being good at manipulating small objects does not necessarily make one good at navigating a city. One corollary to this finding is that processing spatial information at different scales of space relies on different psychological constructs and different physical or functional parts of the brain (Barba & Marroquin, 2017). This paper and the study reported in it concerns the role of large-scale spatial abilities that comes into play when individuals learn an environmental space.

**Fig. 1:**
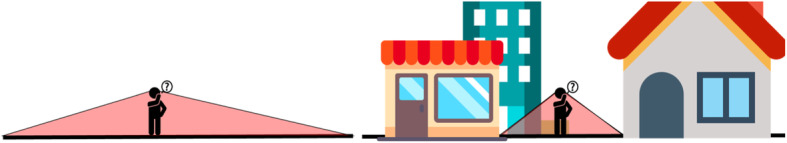
A learner experiences a space from a single viewpoint. The individual visible area, what we refer to as *geographic scale*, is indicated by the semi-transparent red portion. Geographic scale varies with visual barriers, for example being larger in less visually obstructed areas (left) and smaller in more visibly obstructed areas, such as cities (right)

An important characteristic of environmental space is that its spatial properties can be apprehended from direct experience along with prolonged locomotion—that is, the integration of spatial information apprehended from multiple viewpoints perceived by a single individual over time. Here the spatial extent of an environment that learners are able to see from a single viewpoint (geographic scale) comes into play. Some environments, such as empty parking lots or sports fields, have few visual barriers which allow learners to access more information important for spatial learning from a single position and, hence, possess a large geographic scale (Fig. 1, left). In contrast, a viewpoint with small geographic scale covers a small proportion of the total area. In some environmental spaces such as an office with tall partitions or a downtown area with tall buildings, learners’ viewpoints are constrained by visual barriers. As a result, the visible area from a single position tends to be much smaller within such visibly restricted areas, resulting in a small geographic scale (Fig. 1, right). Learners need to locomote to generate an array of viewpoints, requiring the integration of spatial information over periods of time. This paper seeks to further the theoretical understanding of the effects of geographic scale from these viewpoints on the learning process.

There are two ways to manipulate geographic scale: changing the environment to increase visibility from single locations, or changing the perspective of the learner (e.g., Barra, Laou, Poline, Lebihan, & Berthoz, 2012; Restat, Steck, Mochnatzki, & Mallot, 2004; Zhao & Klippel, 2019). Previous studies from psychology and geography have shown that spatial learning can be enhanced by using transparent instead of opaque environments (Belingard & Péruch, 2000; He, McNamara, & Brown, 2019; Piller, 2006; Piller & Sebrechts, 2003). For example, Piller (2006) examined how the spatial layout of a virtual building can be learned by making the walls along the route transparent in which participants could see through the walls but could not pass through them. Participants navigated one of the virtual environments (VEs), opaque or transparent, learning both the route and the locations of 21 objects located in seven rooms. Learning was assessed through target location estimates and map drawings. Results from the study show that compared to the opaque, naturalistic environment, participants who were trained in the transparent environment showed more rapid learning of survey knowledge, and quality of map drawings indicated that the transparent VE led to a more integrated spatial mental model than did opaque navigational learning. In a more recent study, He et al. (2019) investigated the effects of barriers on spatial learning and memory in a virtual shopping mall which were compartmentalized by doors, walls, and buildings. Environment visibility was enhanced by granting participants *X-ray* vision during spatial learning (i.e., all or a subset of buildings that the participant was looking at became transparent). They found that compared to the participants who learned with naturalistic environment visibility, participants in the transparent environment had better performance in wayfinding and pointing tasks; however, these benefits were only observed in participants with high self-report sense of direction. He’s study provides evidence to support the *ability-as-enhancer* hypothesis proposed by Mayer and Sims (1994), which states that in multimedia learning (here: learning from a 3D VE), high spatial ability learners should benefit from rich external representations more (here: the direct relational information about buildings that was disrupted by barriers) because they have enough cognitive capacity left for mental model construction (see also Huk, 2006).

**Fig. 2:**
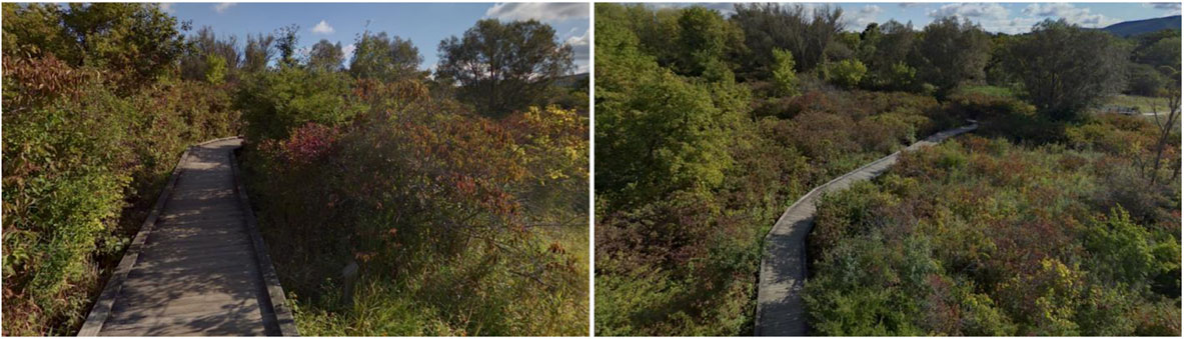
Geographic scale varied by the elevation of the point of view. The left image was taken at normal eye level in Millbrook Marsh Nature Center, PA, USA, while the right image was taken at the same location but at 27 ft./8.2 m above ground

In addition to making walls and buildings transparent in order to lift constraints on the representation, geographic scale can also be manipulated by changing the perspective of the learner. We are used to experiencing the world around us from a ground perspective, but what if we elevate our viewpoint as if we were a giant like the biblical Goliath? Figure 2 shows what this means in terms of being able to access an environment via different geographic scales. From an elevated perspective, a learner has access to a larger number of entities and their relations through direct observation, reducing the need to hold that information in memory. Offloading information into the environment has long been deemed critical for the efficient processing of information by the human cognitive system (Norman, 1993; Raubal & Worboys, 1999). Storing information is seen as expensive, while reading information directly from the environment is comparatively cheap (Clark, 1989; Simon, 1956). Increasing the spatial extent that is directly accessible from a learner’s egocentric perspective through an elevated perspective should unravel more global characteristics from a single viewpoint and hence allow for offloading the understanding and storing of relational information into the environment. This aspect of spatial learning in VEs has yet to be examined.

It is important to note that exploring an environment at elevated level differs from map-based spatial learning, which has been more widely studied in literature (Evans & Pezdek, 1980; Shelton & McNamara, 2004; Shelton & Pippitt, 2007; Snyder, 1998; Thorndyke & Hayes-Roth, 1982; Török, Nguyen, Kolozsvári, Buchanan, & Nadasdy, 2014; Yamamoto & Degirolamo, 2012). A basic distinction is that maps are a form of representation abstracted to varying degrees from the real environment, while elevated views are comprised of real or simulated *direct* sensing of the environment by an observer. Several more practical differences are related to this distinction. First, while the elevated perspective offers access to a larger geographic scale than the normal ground perspective, learners from the elevated perspective still need to move within the environment to perceive the spatial layout of the environmental space. In contrast, a map is normally much smaller than the space it represents, from which learners can obtain survey-like knowledge of the environment from a single viewpoint (i.e., figural or vista space). Second, unlike a map in which the space represented typically has a fixed cardinal direction,^1^ learners from the ground or the elevated perspective turn their heads and bodies to change looking directions. Third, learners from the elevated perspective explore an environment with an oblique angle of view, whereas for map reading the view angle is orthogonal. Finally, unlike the normal ground view or map reading, experiences at elevated level are rarely available in everyday life. Table 1 summarizes this brief discussion.

**Table 1:** Comparison of the Ground Perspective, the Elevated Perspective, and Map Reading for Spatial Learning

	Ground	Elevated	Map
Geographic scale	Small/Local	Medium	Large/Global
Orientation agency	✓	✓	Seldom
View angle	Flat	Oblique	Orthogonal
How often experienced	Everyday	Rarely	Often

An important feature of spatial learning, as proposed by Freksa and Barkowsky (1996), is the ability to switch between views. As human beings, it is impossible to make all potentially interesting aspects of the world simultaneously explicit within one representation medium (e.g., a map). In order to form a global understanding of the environment, our different aspects of interest that were acquired at different time points need to be compatible and fit into one reference system corresponding to a global view over significant periods of time. Assembling these aspects of the space that are represented separately is deemed expensive since a lot of implicit information must be integrated to build relations. Freksa and Barkowsky (1996) suggest that an environmental space can be understood as a discontinuous entity composed of a series of inherently discrete views. Each single view represents a certain area or spatial extent of the space, which is often constrained by the normal eye level of the learner. The accuracy of a mental representation of an environmental space constructed from a series of discrete vistas may largely depend on the individual’s spatial ability. Understanding how a space can be described and learned based on these discrete views is the key to formalize and quantify the spatial learning process.

### The current study

In an attempt to address the particular aforementioned aspects of spatial learning we focus on how geographic scale influences spatial learning while learners experience discrete viewpoint transitions (i.e., teleportation; see Weißker, Kunert, Frohlich, & Kulik, 2018 for review) in an environmental space. We conducted two experiments investigating the effects of experiencing a VE composed of 360° images^2^ at different geographic scales. We compared navigating an environment through pseudo-aerial 360° images taken at a height of 17.5 ft./5.3 m (elevated perspective) to 360° images taken at 4.5 ft./1.4 m above ground (ground perspective). In Experiment 1, participants were teleported to learn the VE at ground level alone or from ground *plus* elevated perspectives. In Experiment 2, the perspective of participants was confined to a single perspective at all times (ground or elevated).

Learning outcomes were measured using onsite pointing tasks and self-reports of mental workload. Additionally, we included a measure of self-reported sense of direction, which is highly correlated with spatial knowledge acquisition from direct experience in environmental spaces and largely independent of small-scale spatial abilities (Hegarty, 2002; Hegarty et al., 2006). We used absolute pointing errors as well as an analysis of systematic errors to draw conclusions about participants’ learning performances. Pointing error was measured as the absolute angular difference between the judged pointing direction and the actual direction of the target.^3^ The systematic error analysis probes whether errors could be specifically modeled as non-random deviations from the correct pointing direction, such as on the basis of wrong turns or forgetting segments (Meilinger, Henson, Rebane, Bülthoff, & Mallot, 2018). Because a learner with access to a larger geographic scale can be freed from the constraints of the limited spatial extent of the environment accessible through experiences at normal eye level of the learner, we hypothesize that learning from the elevated perspective yields smaller errors in the onsite pointing task than at ground level alone. In contrast to our hypothesis, the results of the pointing and systematic error analysis show no significant effects of perspective on spatial learning but suggest a facilitatory role of elevated perspective in bridging the performance gap between low and high spatial ability learners. Discussing the results, we detail our efforts to systematically address the effects of scale and examine the conditions under which spatial learning would be maximally effective.

## Experiment 1

### Method

#### Participants and design

**Fig. 3:**
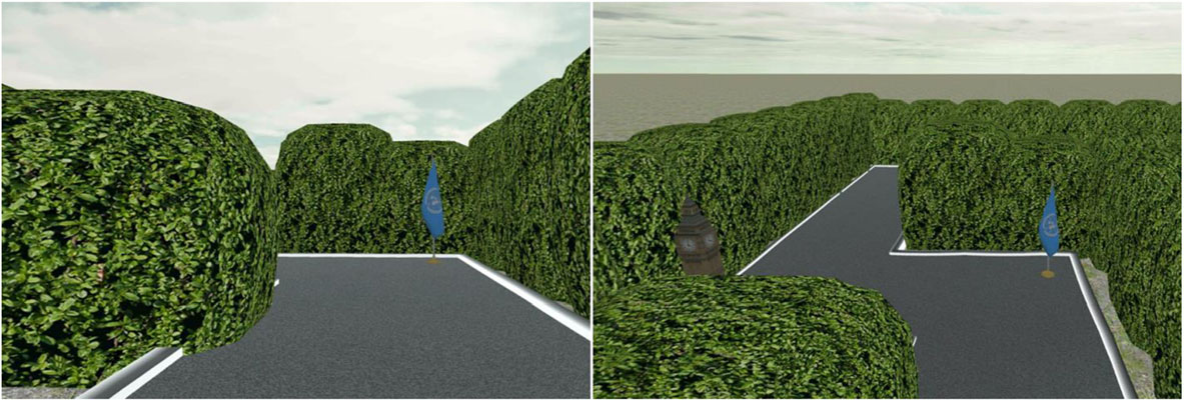
Change of geographic scale at a single position within a virtual maze. Left: ground perspective (4.5 ft./1.4 m above ground); the blue flag was the only visible landmark. Right: elevated perspective (17.5 ft./5.3 m above ground); both the blue flag and Big Ben could be seen from a single viewpoint. The spatial extent visually accessible from a single viewpoint was controlled by hedges along both sides of the path

We recruited 51 students from The Pennsylvania State University. Due to simulator sickness (1 participant) and errors in the visible pointing trials (2 participants whose average visible pointing errors were larger than Q_3_ [75th quartiles] + 1.5xIQR [interquartile range]; see the Procedure and Measures section for more details), we ended up with 48 participants (21 females) with ages ranging from 18 to 30 years (*M* = 21.6 years, *SD* = 3.08). The experiment employed a between-subjects design, in which participants learned a virtual maze from one or two perspectives (Fig. 3). Participants were randomly assigned to the ground (G) group (24 participants, average age 21.4 years, 11 females), in which their viewpoints were confined to a ground level (4.5 ft./1.4 m above ground) at all times, and to the ground + elevated (G + E) group (24 participants, average age 21.8 years, 10 females), in which they were offered access to an elevated 17.5 ft./5.3 m high perspective in addition to the normal ground perspective. All participants were financially compensated for their participation.

#### Materials

##### The virtual maze

**Fig. 4:**
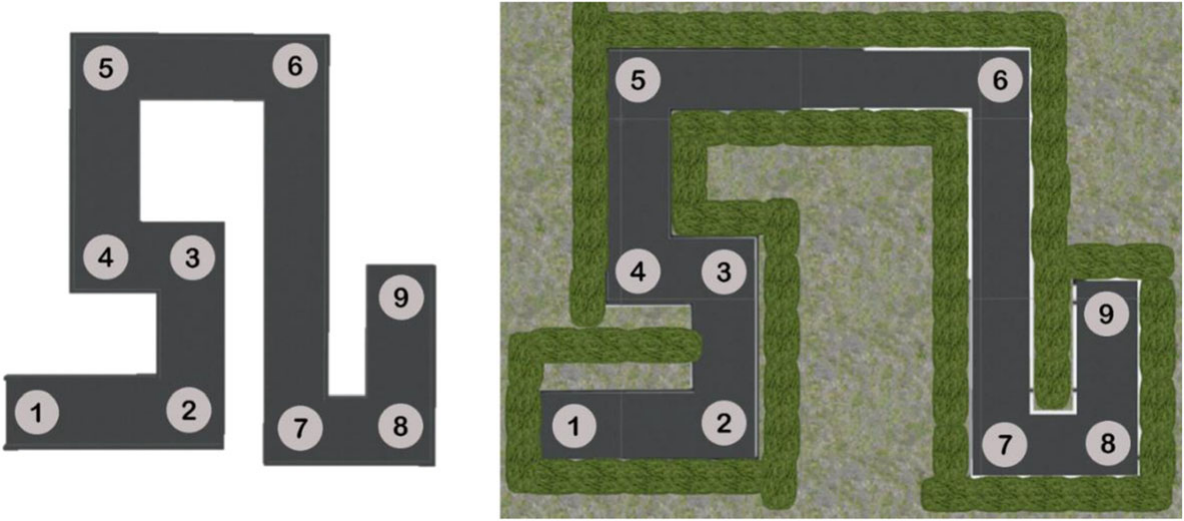
Aerial views of the original route layout (left; Meilinger et al., 2018) and environmental layout of the virtual maze (right). Circles represent testing and target locations for the onsite pointing task. The circles are numbered from start across all turns to the end of the route

The VE used in this experiment was adapted and modified from Meilinger et al. (2018). The original was designed to examine the mechanism that human users applied to acquire survey knowledge. In our version, the layout was modified to precisely manipulate geographic scale, for example, by extending some distances between landmarks to reduce intervisibility. Figure 4 shows a planar view comparison of the original maze used in Meilinger’s study and our virtual maze. The route of the virtual maze consisted of a start and endpoint, as well as seven turns along the route. These nine testing locations were rendered using high-resolution 360° images taken within the virtual maze and were associated with nine salient landmarks: Lighthouse, Traffic Light, Blue Flag, Big Ben, Chimney, Street Lamp, Statue of Liberty, Payphone, and Eiffel Tower. While all environmental landmarks had a columnar shape in order to reduce systematic errors in the onsite pointing task, they were distinct in structure and texture to uniquely distinguish locations. A pilot study as well as the results discussed here confirmed that there was no systematic bias toward any of the landmarks.

##### The experimental setup

The virtual reality setup consisted of a stand-alone Oculus Go^4^ head-mounted display with its synchronized handheld controller offering 3DOF orientation tracking. The virtual content was rendered using the Unity3D^5^ game engine with a display refresh rate of 60 - 72 Hz. Participants were seated in swivel chairs which allowed for turning head and body to perceive vestibular feedback. Participants viewed the 360° images of each location with a field of view of 101° at a display resolution of 2560 × 1440 pixels.

#### Procedure and measures

After consenting, participants provided basic demographic information (e.g., gender, age, and ethnicity) and completed the Santa Barbara Sense of Direction Scale (SBSOD) through Google Forms. The SBSOD is a self-report measure of spatial and wayfinding abilities, preferences, and experiences in everyday navigation activities (Hegarty, 2002). They then wore an Oculus Go headset and familiarized themselves with the interactions required. Thereafter, participants were randomly assigned to the ground (G) or ground + elevated (G + E) group and experienced two test sessions. Each test session involved a 6-min passive exposure to the virtual maze and the onsite pointing task and concluded with the NASA Task Load Index (TLX), all further detailed below.^6^ Participants took a three-minute break between sessions. The whole experiment lasted approximately one hour.

##### Passive exposure to the virtual maze

In the learning phase, participants were instructed to learn the locations of nine landmarks in the virtual maze. They navigated the maze by being teleported instantaneously to each location in order and then passively retracing the route in reverse upon reaching the end. This gave them nine discrete viewpoints, rendered by 360° images, at the testing locations along the route. In this phase, participants experienced passive exposure to the virtual maze, a system-guided tour that forced the transitions of viewpoints. Participants in the G group, whose viewpoints were constantly placed at 4.5 ft./1.4 m above ground, were offered 20 s to learn each testing location. In addition to the ground perspective, participants in the G + E group had access to an elevated 17.5 ft./5.3 m high perspective at locations 2, 4, 6, and 8 (see Fig. 4), where one additional landmark can be seen from the elevated perspective compared to the ground perspective. To control for the total time spent in the learning phase (six minutes), each of these locations allowed for an exposure of 27 s, while the exposure time at ground-only locations (locations 1, 3, 5, 7, and 9) was reduced to 15 s for this group.

##### Onsite pointing task

In the following testing phase, participants wore an Oculus Go headset and were tested on how well they had learned the spatial relations between landmarks in the virtual maze (Meilinger et al., 2018). Participants were randomly teleported to a testing location and were instructed to point to the remaining target locations one by one, during which their perspective was confined to the ground level, and the name of the target location was displayed on the display for each pointing trial (e.g., *“Point to the payphone”*). Once finished, participants were randomly placed at a new position and repeated the pointing trials for the remaining eight locations. In total, 72 directions along with pointing errors were recorded. The direction records were divided into two groups: *visible pointings* where the testing and target locations were intervisible from a ground perspective, and *non-visible pointings* where the lines of sight were blocked by hedges between the testing and target locations. These resulted in 16 visible and 56 non-visible pointings. The visible pointing trials should be very simple to complete and thus served as a reference measure on how accurate participants could become in performing the onsite pointing task.

##### Mental workload

The NASA-TLX for evaluating the subjective experience of workload was measured using a Google Forms questionnaire (Hart & Staveland, 1988). It consists of six subscales describing *mental demand*, *physical demand*, *temporal demand*, *performance*, *effort*, and *frustration*. The responses on each scale were analyzed separately. We changed the original NASA-TLX continuous rating scale (0–100) to a 10-point scale,^7^ and administered it after the onsite pointing task. An overall estimate of mental workload, NASA Raw TLX, was also calculated by taking the sum of the six TLX components and dividing it by six (Miller, 2001).

### Results and discussion

To examine scale effects in relation to individual differences in spatial abilities (measured through the SBSOD scale), we separated the 48 participants into 24 high sense-of-direction (SOD) and 24 low-SOD participants using the median SOD score for both perspective groups (G and G + E). This split resulted in 11 high- and 13 low-SOD participants in the G group; and 13 high- and 11 low-SOD participants in the G + E group. Pointing errors (measured through average non-visible pointing errors in the two test sessions) and self-reported mental workload (i.e., NASA Raw TLX) were analyzed in generalized linear mixed models (GLMMs). Specifically, perspective group (G vs. G + E), SOD level (low vs. high), and test session (1st vs. 2nd) were modeled as fixed effects of the target (pointing error/mental workload).

**Fig. 5:**
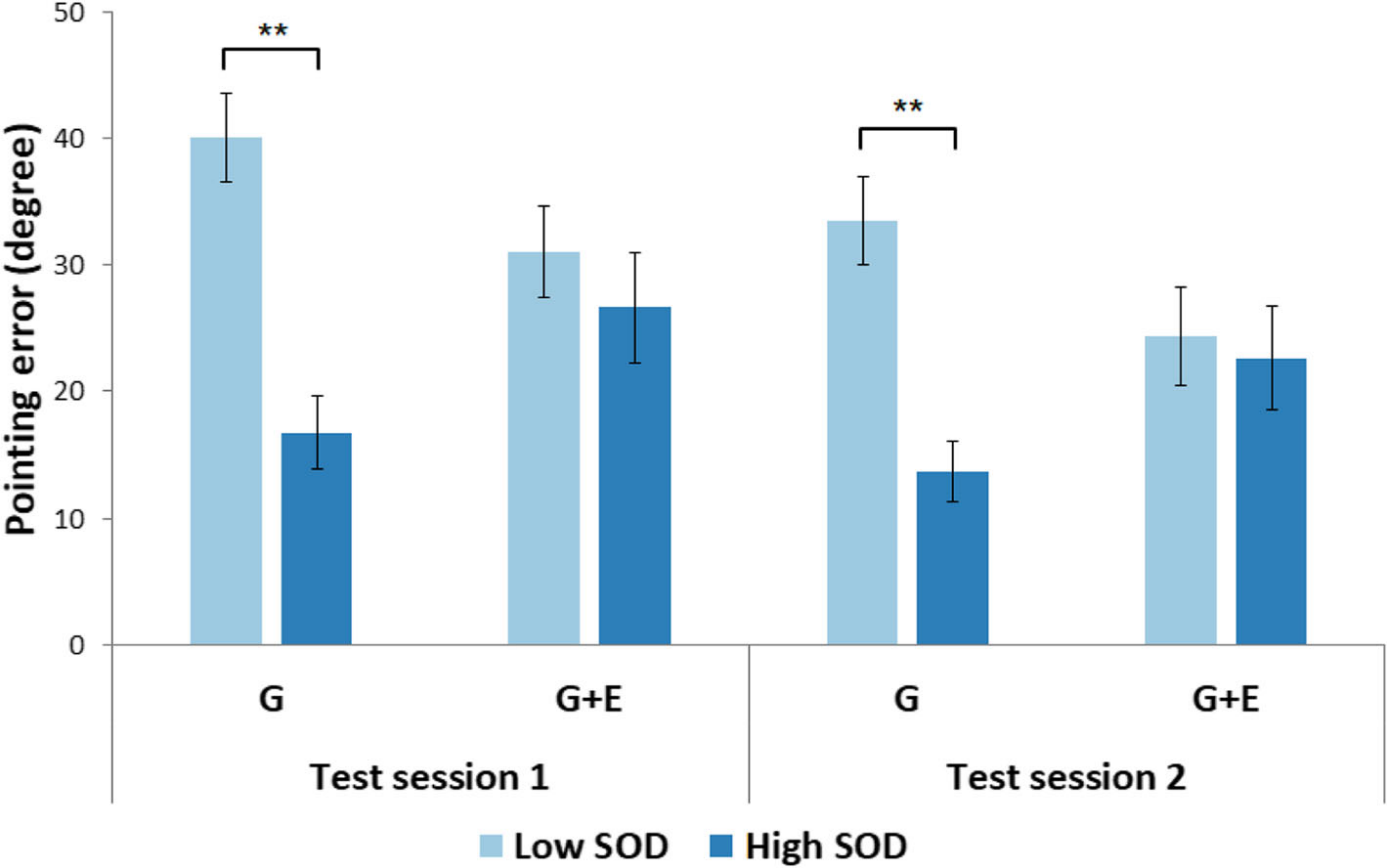
Pointing errors of low and high sense-of-direction (SOD) participants varied across ground (G) and ground + elevated (G + E) groups in both test sessions in Experiment 1. Error bars represent ±1 standard error of the mean. **p < .01

For the pointing errors (Fig. 5), the main effects of the test session, *χ*^2^(2) = 18.75, *p* < .001, and the SOD level, *χ*^2^(2) = 10.43, *p* = .001, were significant. However, the pointing errors made by G and G + E participants were not significantly different, *χ*^2^(2) = .003, *p* = .96. There was a significant interaction effect of the SOD level and perspective group on pointing error, *χ*^2^(2) = 7.06, *p* = .008. This indicates that the main effect of SOD level on pointing errors described previously was different between the two perspective groups. No other two-way interactions were significant, *χ*^2^(2)’s < 2.18, *p*’s > .14. The three-way interaction (test session x SOD level x perspective) was not significant, *χ*^2^(2) = .04, *p* = .84. Pairwise comparisons with Tukey corrections using the *emmeans* package in R (Lenth, 2019) indicate that for both test sessions, low-SOD participants made significant larger pointing errors than high-SOD participants when they were in the G group (1st session: *t.ratio*(44) = 4.49, *p* = .001, *estimate* = 23.29; 2nd session: *t.ratio*(44) = 3.81, *p* = .009), *estimate* = 19.75), while there were no significant differences in the G + E group (1st session: *t.ratio*(44) = .85, *p* = .99, *estimate* = 4.38; 2nd session: *t.ratio*(44) = .33, *p* = 1.0).

For mental workload, there were no significant group differences or interactions (*χ*^2^(2)’s < 2.75, *p*’s > .09) except for the significant main effect of test session, *χ*^2^(2) = 34.04, *p* < .001, which indicates that from the first to the second test session, there was a significant reduction in mental workload (1st session: *M* = 5.64, *SD* = 1.22; 2nd session: *M* = 4.54, *SD* = 1.64), *t*(44) = −7.15, *p* < .001, *r* = .73. Next, we examined the effects of perspective group on the six-component scales of the NASA-TLX using pairwise comparisons with Bonferroni corrections. The only significant difference was that, in the first test session, participants in the G + E group (*M* = 5.08, *SD* = 2) reported significantly higher temporal demands than those in the G group (*M* = 3.42, *SD* = 1.47), *p* = .012. There were no significant differences in the second test session.

The pattern of results for pointing errors indicates that, while there was no significant difference between the two perspective groups, the significant performance difference between the low- and high-SOD participants in the G group was largely diminished when participants had access to the elevated perspective.

## Experiment 2

Experiment 2 was identical to Experiment 1 except that the ground perspective was eliminated from the elevated perspective group (i.e., G + E group in Experiment 1). In other words, participants in both perspective groups experienced a location through a single perspective (ground *only* or elevated *only*). Our conjecture was that the dynamic change of geographic scale would increase time pressure observed in the G + E group in Experiment 1. Studies suggest that time pressure has negative effects on various learning conditions (e.g., Chuderski, 2016; Wilkening & Fabrikant, 2011), which may offset the potential benefits of accessing an elevated perspective to spatial learning. Having participants in an elevated perspective group explore from the elevated perspective *only* may help to diminish the group difference in perceived time pressure.

### Method

#### Participants

Thirty-five students were recruited from The Pennsylvania State University and participated in this experiment in return for extra credit in psychology courses. They were combined with 24 participants in the G group in Experiment 1 for data analysis. Due to errors in the visible pointing trials, we ended up with 31 participants in the G group (13 females), with an average age of 20.6 years, and 28 participants in the elevated (E) group (14 females), with an average age of 21.3 years.

#### Materials, design and procedure

This experiment was identical to Experiment 1 except that participants in the E group experienced passive exposure to the virtual maze from the elevated perspective *only* and were offered 20 s to learn each testing location (same as the G group in both experiments).

### Results and discussion

**Fig. 6:**
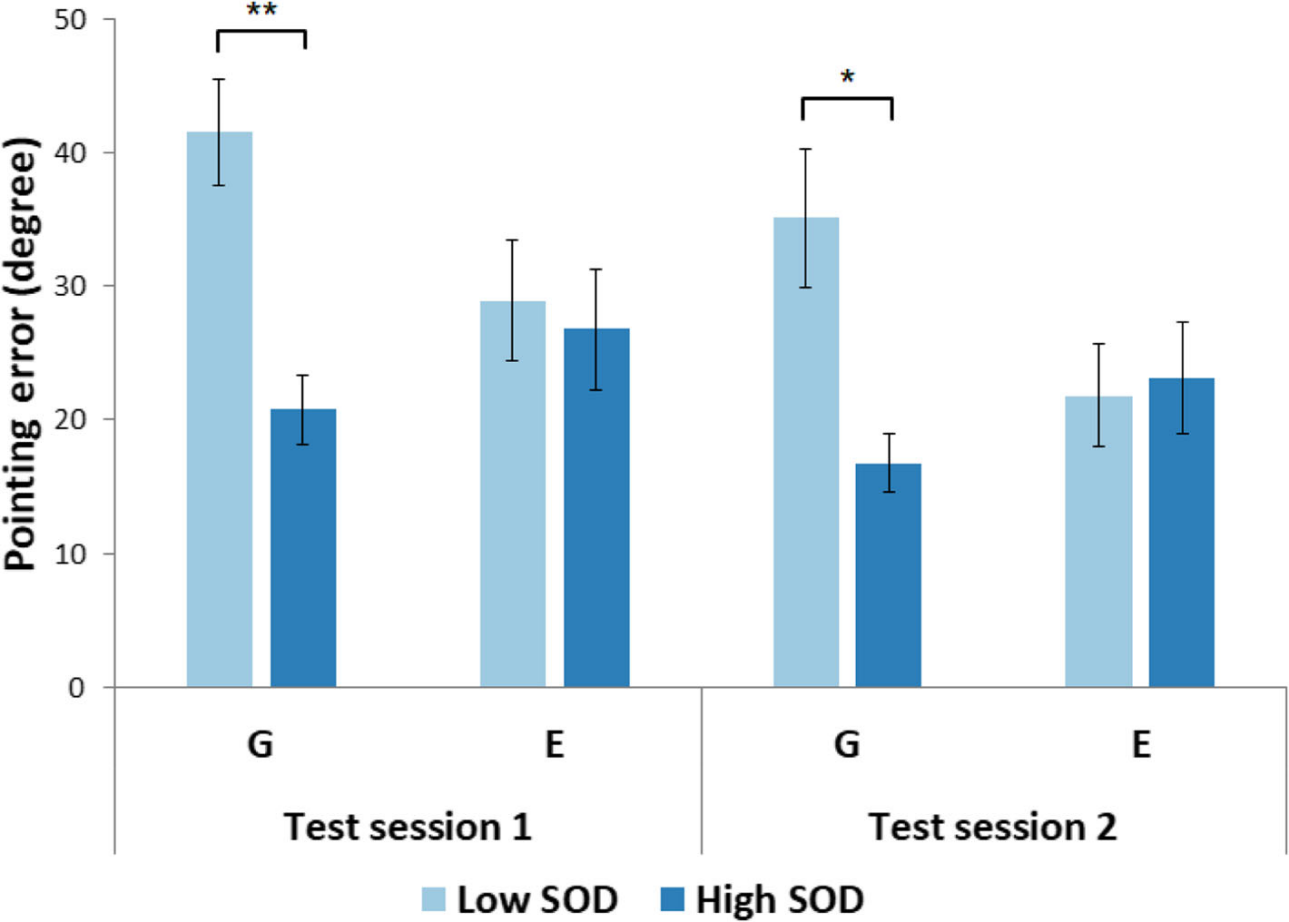
Pointing errors of low and high sense-of-direction (SOD) participants varied across ground (G) and elevated (E) groups in both test sessions in Experiment 2. Error bars represent ±1 standard error of the mean. *p < .05, **p < .01

We analyzed the data in the same way as in Experiment 1. For the pointing errors (Fig. 6), the main effects of the test session, *χ*^2^(2) = 17.65, *p* < .001, and the SOD level, *χ*^2^(2) = 6.85, *p* = .009, were significant. However, the pointing errors made by G and E participants were not significantly different, *χ*^2^(2) = .77, *p* = .38. There was a significant interaction effect of the SOD level and perspective group on pointing error, *χ*^2^(2) = 6.86, *p* = .009. This indicates that the main effect of SOD level on pointing errors described previously was different between the two perspective groups. No other two-way interactions were significant, *χ*^2^(2)’s < 1.60, *p*’s > .20. The three-way interaction (test session x SOD level x perspective) was not significant, *χ*^2^(2) = .05, *p* = .83. Pairwise comparisons with Tukey corrections using the *emmeans* package in R (Lenth, 2019) indicate that for both test sessions, low-SOD participants made significantly larger pointing errors than high-SOD participants when they were in the G group (1st session: *t.ratio*(55) = 3.89, *p* = .006, *estimate* = 20.75; 2nd session: *t.ratio*(55) = 3.44, *p* = .023), *estimate* = 18.31), while there were no significant differences in the E group (1st session: *t.ratio*(55) = .38, *p* = 1.0, *estimate* = 2.12; 2nd session: *t.ratio*(55) = −.24, *p* = 1.0).

For mental workload, there were no significant group differences or interactions (*χ*^2^(2)’s < 1.08, *p*’s > .29) except for the significant main effect of test session, *χ*^2^(2) = 34.04, *p* < .001, which indicates that from the first to the second test session, there was a significant reduction in mental workload (1st session: *M* = 5.3, *SD* = 1.26; 2nd session: *M* = 4.78, *SD* = 1.06), *t*(44) = −7.15, *p* < .001, *r* = .73. Next, we examined the effects of perspective group on the six-component scales of the NASA-TLX using pairwise comparisons with Bonferroni corrections. There were no significant differences, indicating that the group difference of time pressure observed in Experiment 1 has been eliminated (G: *M* = 3.5, *SD* = 1.65; E: *M* = 3.48, *SD* = 1.54).

The only difference between Experiments 1 and 2 was that the ground perspective was excluded from the elevated perspective group in Experiment 2. While this manipulation eliminated the group difference on perceived time pressure, there was no evidence of the advantage of elevated perspective when we compared G with E groups in Experiment 2. Similar to Experiment 1, sense of direction played a significant role in spatial learning through experiences at ground level alone; however, participants with access to the elevated perspective had similar pointing performances regardless of their spatial abilities.

## Error analysis and modeling

In addition to the effect of geographic scale on spatial learning performance as indicated by the absolute pointing error, we wished to probe whether geographic scale and individual differences in spatial abilities influenced the systematic distortion of mental representations by analyzing and modeling systematic errors. The pointing data from Experiments 1 and 2 were merged for this analysis.

### Source of pointing errors

**Fig. 7:**
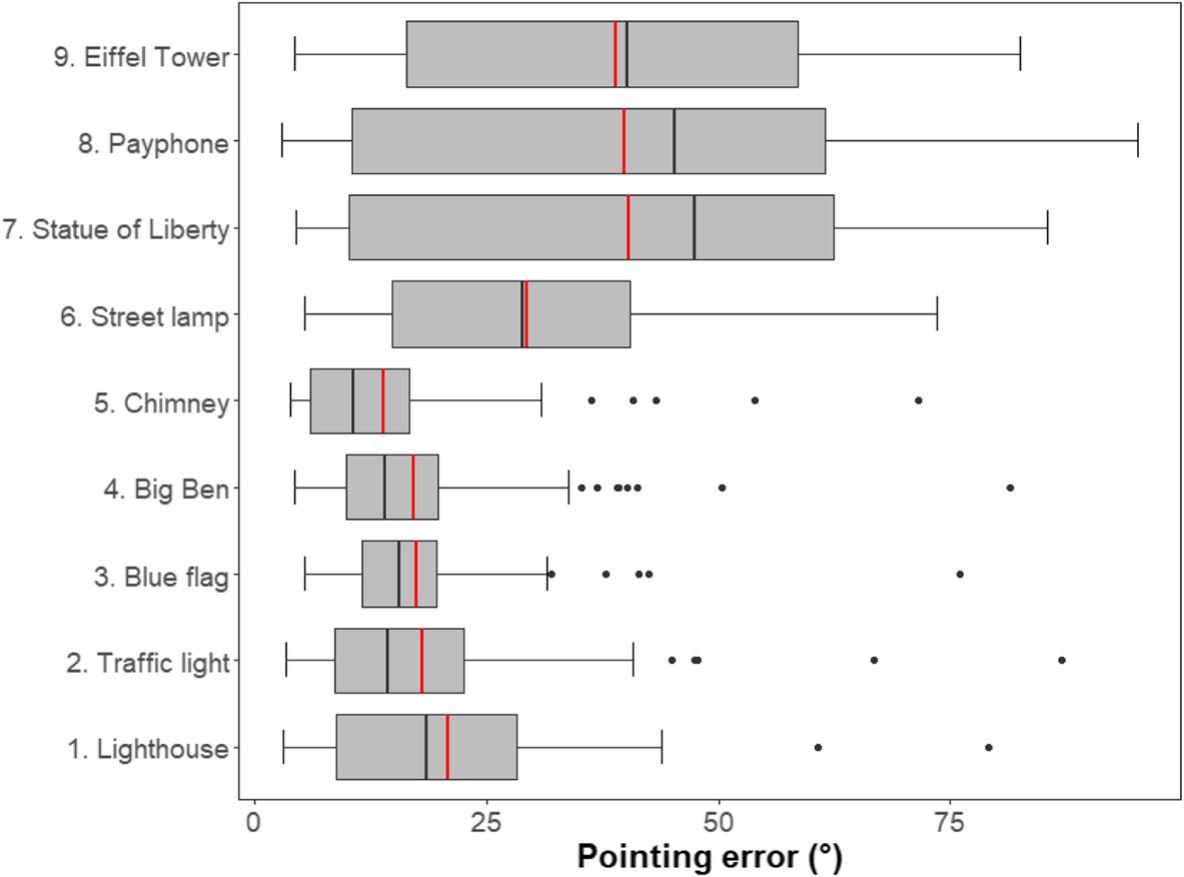
Box plots of pointing errors across pointing trials towards each target location for both experiments. In the box plots, the boundary of the box closest to zero indicates the 25th percentile, a red line within the box denotes the mean, a black line within the box denotes the median, and the boundary of the box farthest from zero indicates the 75th percentile. Whiskers indicates the 10th and 90th percentiles. Points represent outliers outside the 10th and 90th percentiles. The pointing errors made by a single participant were averaged for the same target location. From bottom to top, the target locations are sorted by the order in which they were passed when the participants learned the virtual maze for the first time

Figure 7 shows the pointing errors that participants made toward each target location. Since the assumption of homogeneity of variance was violated, *F*(8, 738) = 35.36, *p* < .001, a Friedman test, as the non-parametric statistical test for repeated measures, was conducted to test whether there was any significant difference in pointing error between pointing groups (nine pointing groups in total corresponding to the nine target locations). There was a significant difference, *χ*^*2*^(8) = 262.71, *p* < .001, *W* = .40. The Friedman test was followed up with pairwise comparisons with Bonferroni corrections, which indicate that, compared to the first five target locations (locations 1–5), participants made significantly larger pointing errors when pointing toward the last four target locations (locations 6–9), *p*’s < .009.

### Systematic error analysis

**Fig. 8:**
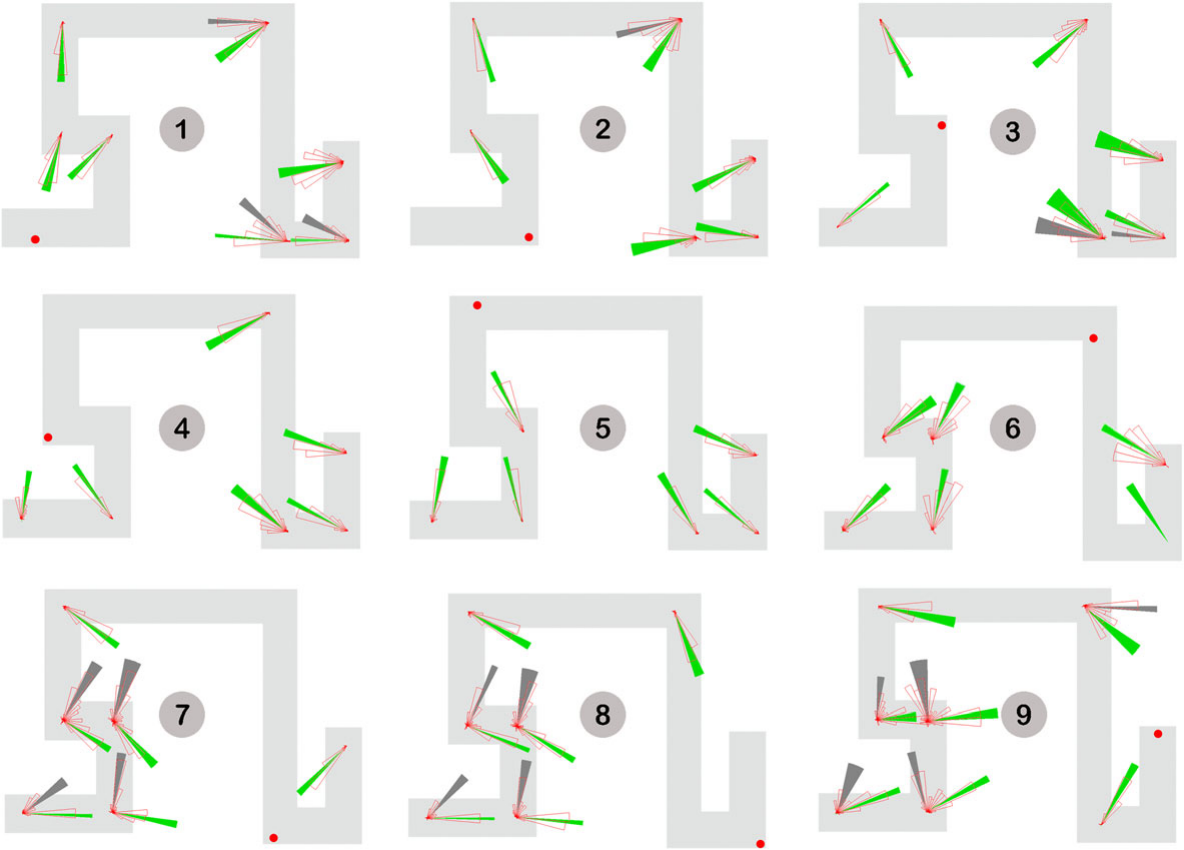
Unimodal or bimodal distributions across non-visible pointing trials. Target locations are indicated by red dots and numbered by gray circles. Circular histograms indicating pointing directions are shown at corresponding testing locations. A green or dark gray sector plots the 95% confidence interval of the average pointing direction for each modal. For bimodal distributions, errors that were modeled as the higher angular deviations from the correct pointing directions were assumed as systematic errors and indicated by the dark gray sectors

The systematic error analysis examines whether pointing errors could be specifically modeled as non-random deviations from the correct pointing direction (Meilinger et al., 2018). In practice, systematic errors are the result of mixing up turns or forgetting segments during mental mapping. Meilinger et al. (2018) suggest that such qualitative errors are mostly characterized by bimodality observed in the pointing data. To identify if there was any underlying bimodal distribution(s) across pointing trials, we performed a Bayesian analysis of directional data from a finite mixture distribution using the *BAMBI* package (Chakraborty & Wong, 2017) in R. Eighteen out of 56 non-visible pointings were identified as being bimodally distributed (Fig. 8), 13 of which occurred when participants pointed to the last three target locations (locations 7–9). Given the fact that pointing to these target locations also yielded significantly increased pointing errors (see the Source of Pointing Errors section), systematic errors, which are indicated by the dark gray sectors in Fig. 8, were deemed an important source of the pointing errors. Note that although there was no bimodality observed in the pointing data for location 6, participants made considerable errors when pointing to this location. Fig. 8 shows that there were clear deviations from the correct direction in at least three pointing trials (i.e., 2 → 6, 3 → 6, and 9 → 6). Such unimodal but deflected distributions might be a result of some quantitative errors, such as distance underestimation due to teleportation, that were incorporated during spatial learning (see further discussion in the General Discussion section).

The systematic error analysis provides evidence that participants’ mental representations driving estimates of direction may be systematically distorted. Notwithstanding, it is important to note that not all spatial knowledge is distorted. Some of it indicated by the unimodal pointings is quite accurate. The inconsistencies agree to the term *cognitive collage* (Tversky, 1993), which emphasizes the fact that mental representations for memory and judgement are partial, fragmented, and hierarchically structured (Mark, Freksa, Hirtle, Lloyd, & Tversky, 1999). Because some of the stored information is erroneous, it is unlikely that the pieces of information can be organized into a single, coherent maplike cognitive structure (Tversky, 1993). Therefore, we do not expect to know what the participant’s cognitive map actually looks like; instead we wish to answer the question of which parts of the mental representation of the virtual maze are systematically distorted and how they influence spatial learning.

### Alternative maze model

**Fig. 9:**
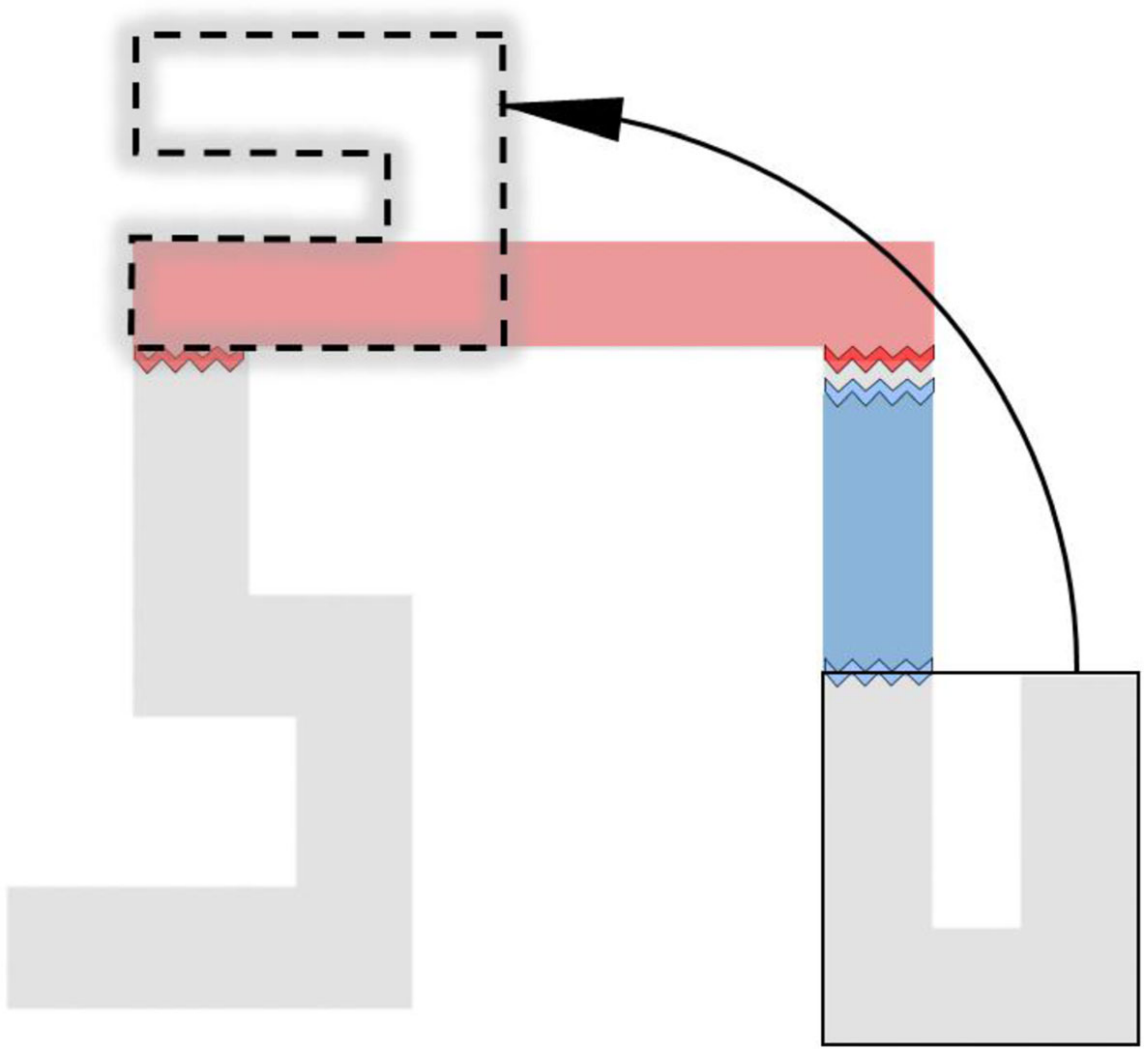
The alternative maze model containing the identified systematic errors generated through 1) eliminating the leg between locations 5 and 6 (i.e., red segment), 2) shortening the leg between locations 6 and 7 (i.e., blue segment) to make it equal to the length of the leg between locations 8 and 9, and 3) rotating the maze component that contained the last three target locations 90° and connecting it to the rest of the maze (illustrated by the arrow)

**Fig. 10:**
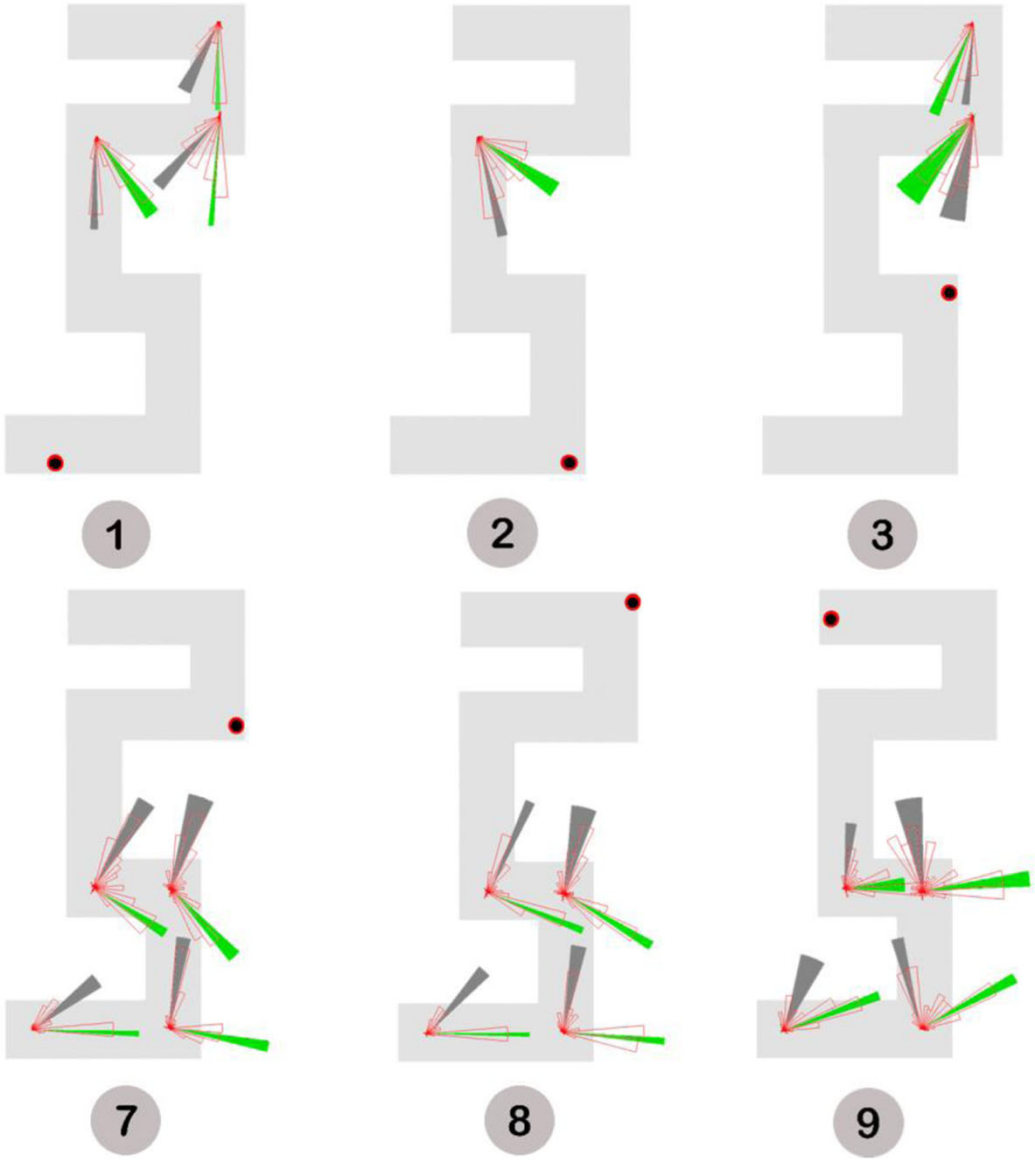
Alternative maze model and bimodal pointing distributions. The black and red dots denote the pointing location assuming participants made systematic errors. The actual target locations are numbered by gray circles. Circular histograms indicating pointing directions are shown at corresponding testing locations. Solid sectors plot the 95% confidence interval of the average pointing direction for individual modals. Specifically, dark gray sectors represent modals that contain systematic errors

In order to fit the identified systematic errors, a single unified representation was generated on the basis of at least three types of errors, including 1) forgetting segments, 2) adjusting leg length (alignment), and 3) estimating on the basis of a smaller number of turns (Fig. 9). As can be seen in Fig. 10, most systematic errors identified in the Systematic Error Analysis section appear to be fitted well into this alternative maze model. Note that the model was estimated from our observations. As detailed in the Limitations and Future Directions section, we are in the process of designing a more formal approach for assessing the fit quality of multiple underlying representations of the virtual maze.

### Error modeling

**Fig. 11:**
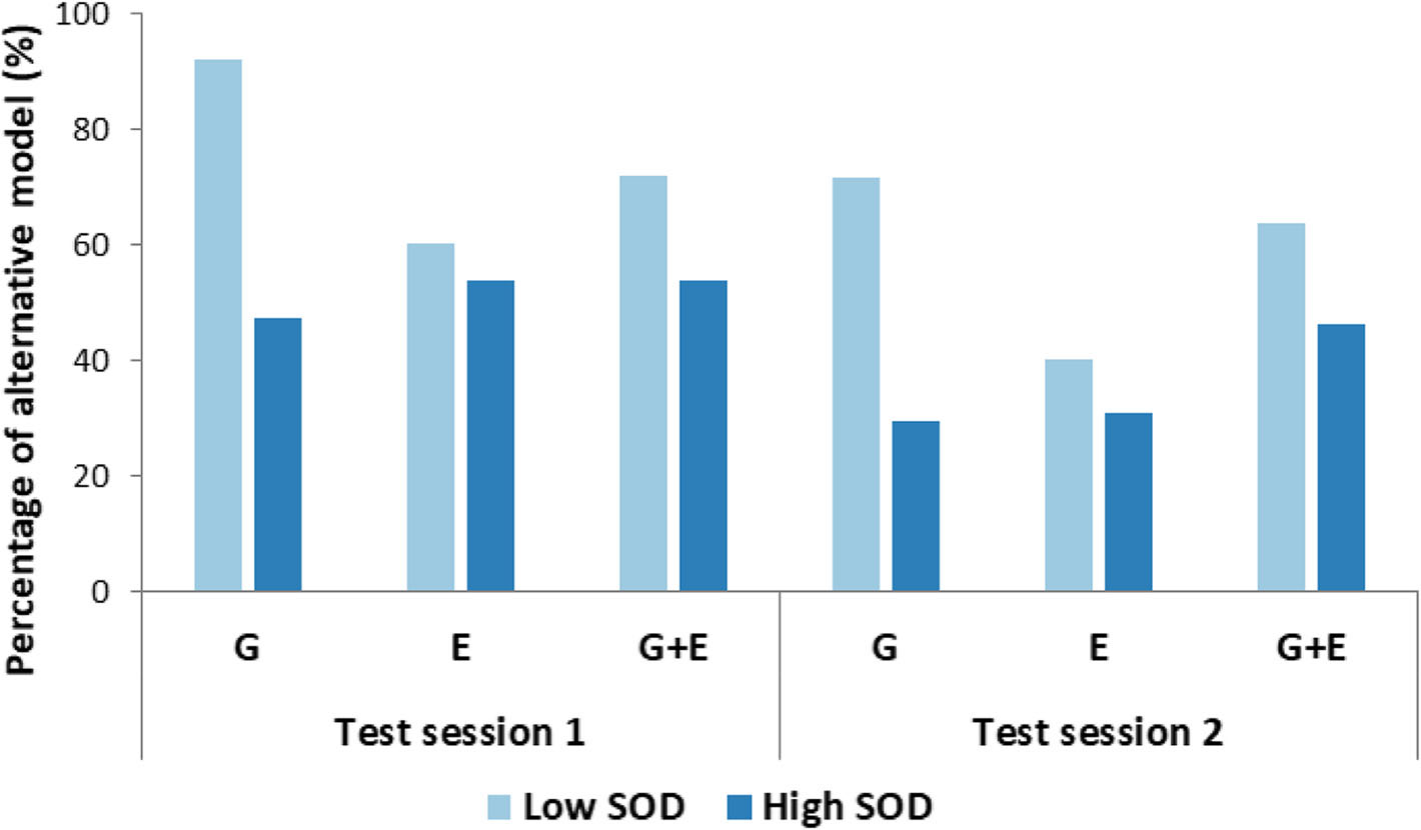
Percentage of low and high sense-of-direction (SOD) participants for which the alternative maze model better fits the pointing data varied across ground (G), elevated (E), and ground + elevated (G + E) groups in both test sessions

To further investigate whether these systematic errors originated from the alternative maze model we compared correct vs. alternative maze representations. For each participant and test session the pointing errors were averaged across pointing trials that contained bimodal distributions. The pointing errors were either derived from the pointings that participants made in the virtual maze or recalculated based on the spatial layout of the alternative maze model. The two maze models were then tested for better fit per participant and test session on the basis of smaller average pointing error. Both perspective group and SOD level were included for analysis (Fig. 11).

Overall, 62.7% of participants in test session 1 and 45.75% of participants in test session 2 were inclined to the alternative maze model. Fitting the alternative maze reduced the pointing errors in average by 62.8% for test session 1 and 62.4% for test session 2.

In both test sessions, compared to the E and G + E groups, the G group had the largest differences in the percentage for the alternative model between the low- and high-SOD participants, indicating that the elevated perspective can help bridge the performance gap between the low- and high-SOD participants while controlling for systematic errors.

In each test session, the alternative model was the better fit for a similar percentage of participants in the E group and high-SOD participants in the G group, which was much smaller than that of low-SOD participants in the G group. In other words, low-SOD participants were likely to benefit more from the elevated perspective than high-SOD participants.

From the first to the second test session, the reduction in the percentage of observing the alternative model in the G + E group (13.3%) was considerably smaller than in other perspective groups (G: 28.57%; E: 37.5%). It is possible, compared to participants in the G and E groups, that the higher time pressure reported by G + E participants in test session 1 might have interfered with the later learning phase, and hence had negative effects on their pointing performances in test session 2.

## General discussion

The current study investigated how scale affects spatial learning in an environmental space. We conjectured that learners would benefit from an increased geographic scale through experiences at an elevated perspective. The combined results from Experiments 1 and 2 indicate, contrary to our expectations, that the effects on participants’ pointing errors are not as strong as hypothesized when we compare the ground + elevated (G + E) group or the elevated group (E) with the ground (G) group. In the G + E group, participants had access to both 4.5- and 17.5-ft high perspectives at four different locations. At these locations, G + E participants had to transition between perspectives, while the perspective of participants in the G or E groups was confined to either ground or elevated level at all times.

Given the equivalent exposure time between perspective groups controlled by passive exposure in the learning phase, it is not surprising that participants in the G + E group felt significantly higher time pressure than those who only experienced a location through a single perspective. An increased time pressure could conceivably offset the potential benefits of elevated perspective to spatial learning, and hence result in equivalent pointing performance between the G and G + E groups in Experiment 1. While the group difference of time pressure observed in Experiment 1 was eliminated by excluding the ground level from the elevated perspective group in Experiment 2, participants made similar pointing errors across all perspective groups (G, G + E, and E); however, the follow-up systematic error analysis provided evidence that time pressure could have negative effects on spatial learning as indicated by the higher percentage for the alternative model in the G + E group than in the E group in test session 2.

The virtual maze has a simple geometric shape with an orthogonal layout in which salient landmarks are associated with turns along the route. In the study of Meilinger et al. (2018) from which our maze was adapted, learners walked through the VE 20 times and performed a pointing task after every four walkthroughs. In contrast to walking, which provides continuous visual flow, participants in the current study were seated in swivel chairs and teleported through the virtual maze. While the importance of receiving continuous visual flow during spatial learning has been emphasized by previous research (e.g., Bhandari, MacNeilage, & Folmer, 2018; Ruddle, 2013), for participants in the current study, their pointing performance did not differ from those in Meilinger et al. (2018) study. The comparable pointing performance implies that, when learners navigate a simple geometric VE, teleportation may be sufficient for effective spatial learning, which is in line with findings reported by Weißker et al. (2018). In their study, participants applied either continuous visual motion or teleportation to learn a simple street-like VE composed of three orthogonal segments; no performance differences were found in the pointing task. The lack of difference in pointing performance between perspective groups suggests that the simple geometric shape of the virtual maze may compensate for the loss of continuous visual flow caused by teleportation. Consequently, participants could efficiently learn the virtual maze through teleportation at normal ground level alone and were less positively affected by the additional elevated perspective. On the other hand, the systematic error analysis tells a different story: The alternative maze model shows that severe distortions occurred in the leg between locations 5 and 6 and the leg between locations 6 and 7 (Fig. 9). This finding is not surprising in light of Cohen’s (1996) definition of spatial memory as consisting of two major functions: remembering locations and the placement of objects within locations, and remembering how to navigate to and within them. In other words, a complete mental representation should preserve inter-object relationships as well as routes between them. Given the functional role of routes in connecting discrete locations and the fact that the two legs between locations 5 and 7 are the longest segments in the virtual maze, when traversing these legs through teleportation, participants had to put more effort into processing and integrating local pieces of spatial information.

Our study further examined pointing performance separately for low- versus high-SOD participants in the G, G + E, and E groups. Both the absolute pointing errors and systematic error modeling were included in our analysis. The results of Experiments 1 and 2 provide reliable and consistent evidence that the elevated perspective can bridge the performance gap between low and high-SOD participants in spatial learning (see Fig. 5 for Experiment 1 and Fig. 6 for Experiment 2). The follow-up systematic error analysis and modeling (Fig. 11) produce a similar pattern of results and further show that high-SOD participants in the G group and high- and low-SOD participants in the E group yielded similar percentages for the alternative model, all of which were considerably smaller than that of low-SOD participants in the G group. This finding indicates that low-SOD participants seemed to benefit more from the elevated perspective compared to high-SOD participants, which does not support the ability-as-enhancer hypothesis (Huk, 2006; Mayer & Sims, 1994) but instead hints at potential support for the *ability-as-compensator* hypothesis (Höffler & Leutner, 2011; Mayer, 2001) found in studies of digital learning via desktop computers (e.g., Lee & Wong, 2014). In contrast to the ability-as-compensator hypothesis suggesting that high spatial ability learners in particular benefit from explicit graphical presentations (e.g., He et al., 2019), an ability-as-compensator effect posits that it is the low spatial ability learners who profit from rich external representations, as a “cognitive prosthetic” (Jamieson, Cullen, McGee-Lennon, Brewster, & Evans, 2014) that helps them to build an adequate mental model. In the current study, an elevated perspective might act as a “cognitive prosthetic” for participants with low sense of direction; that is, low spatial ability learners could gain a particular benefit from accessing an elevated perspective as they have difficulty mentally constructing their own representation when experiencing the environment at ground level alone. The larger geographic scale offered by the elevated perspective reveals broader sets of spatial relations to foster learning (Freksa & Barkowsky, 1996). The explicit presentation of a larger spatial extent of the environment may offload relational information into the environment, thus reducing the need for storing and processing spatial information (Clark, 1989; Norman, 1993; Raubal & Worboys, 1999; Simon, 1956). Such an interpretation is also in line with the *supplantation theory* proposed by Salomon (1979), which states that an insufficient ability (here: sense of direction) can be supplanted by instructional design (here: the access to an elevated perspective with increased geographic scale depicting a larger spatial extent of the environment). On the contrary, for the G group in which participants’ viewpoints were confined to the ground level at all times, the learning efficiency of low-SOD participants was largely hindered by the reduced geographic scale (denoted by their considerably larger absolute errors and higher percentages for the alternative maze model than for the other groups) because they needed to recognize individual locations mentally while at the same time organizing, comparing, and integrating different parts of the virtual maze into a mental representation (Freksa & Barkowsky, 1996; Montello, 1993).

In contrast to low-SOD participants, high-SOD participants did not benefit from the elevated perspective in terms of pointing performance or percentages for the alternative model. There are potential explanations of this finding, including the theory that high spatial ability learners have a more expansive set of processing strategies for spatial relations (Freksa & Barkowsky, 1996; Lee & Wong, 2014). Their working memory needs to attend only to a few elements in order to hold and store most of the to-be-learned information. Consequently, their ample cognitive resources may be more open to distraction, namely elements that were irrelevant to the task such as the skybox or hedge texture (contextual immersion effects caused by *seductive details*; see Lan, Fang, Legault, & Li, 2015 for review), which in turn degraded their task performance. Additionally, participants’ spatial ability was measured using the SBSOD scale. This self-report measure is designed for assessing spatial ability that reflects the sense of direction in everyday navigation activities, implying experiences through a normal perspective at ground level. It is not clear how the SOD comes into play when the spatial learning process involves a novel elevated perspective. Given the probability that the spatial ability measure used in our study is optimized for ground level perspectives, an additional elevated perspective may provide redundant information to high spatial ability learners, thus imposing extra cognitive load for them and breaking up their mental map construction.

The results are also consistent with evidence from the study of Yamamoto and DeGirolamo (2012) investigating the effect of aging on spatial learning from the ground or aerial perspective, which indicates a particular benefit of map reading for older learners. In this study, young and older participants encoded landmarks in a VE either through a ground perspective or map reading. Compared to young adults, older adults experienced certain degrees of decline in spatial learning abilities (Devlin & Wilson, 2010; Lokka & Çöltekin, 2020; Yamamoto & Degirolamo, 2012). When participants were later asked to reconstruct the spatial layout, older (or low spatial ability) participants were less accurate than young (or high spatial ability) participants in the ground, but not the map, encoding condition. With regard to the elevated perspective used in the current study, although it is considered different from map reading according to Table 1, it is possible that they affect spatial learning in a similar manner as both allow learners to comprehend spatial relations between objects as seen from above with an increased geographic scale. Future study is necessary to clarify the specific contribution of elevated perspective with respect to map reading to the improvement of spatial knowledge acquisition.

Taking individual differences into account offers another explanation of the mixed effect of the elevated perspective on spatial learning: the dual representation system of spatial exploration and navigation. Colle and Reid (1998) propose a dual-mode model of spatial learning in an environmental space. This model consists of two modes: a *gaze viewing mode* and a *route tour mode*. In the gaze viewing mode, the learner acquires a spatial representation of a local or vista space that is within his/her spatial span of attention from a single viewpoint. Representations of vista spaces are obtained perceptually while the learner moves within the environmental space, from which a global survey representation of the environmental space is developed. Object locations in this representation are encoded such that their relative positions are maintained in the representation, as are Euclidean distances and angular directions between the objects. The route tour mode, on the other hand, leads to representations that are topographically organized (Piller, 2006). Representations in this mode are obtained via the routes that are navigated from vista space to vista space. The learner does not develop a precise representation of the relative positions of objects within the environmental space, but instead obtains knowledge about how to get here from there in terms of actions that need to be taken, by defining turns and decision points.

Several studies in spatial navigation have demonstrated pronounced individual differences in the use of different navigation modes or strategies to encode spatial information (Aginsky, Harris, Rensink, & Beusmans, 1997; Goeke et al., 2015; Piller, 2006; Weisberg & Newcombe, 2016); therefore, it is important to understand the influence of using particular modes on spatial knowledge acquisition. Considering spatial learning from the ground versus elevated perspective, individual proclivities to use a gaze viewing or a route tour mode can bias mental representations of the environmental space, either producing enhancements or deficits on various measures of spatial learning. If learners are inclined to use the gaze viewing mode, the elevated perspective may in particular show a benefit for their spatial learning, because the larger spatial extent accessible from the elevated perspective leads to less representations of vista spaces that are needed to develop a global representation of the virtual maze. In contrast, when the route tour mode is preferred, the additional spatial information offered by the elevated perspective becomes less beneficial to and may even distract learners from the acquisition of route knowledge. An obvious question for future research would be to consider the interaction effect of the perspective group and dual representation system on spatial learning in an environmental space.

Learning in many disciplines, such as geography and geosciences, relies on place-based learning through field trips or site visits, which require integrating knowledge of phenomena in environmental spaces (Zhao & Klippel, 2019). Due to the rugged terrain and trees, in many cases not all regions of a field site are readily available to students; locomotion is required to link localized and limited spatial relations into a psychological whole. Thus, students with lower large-scale spatial skills may struggle to develop an adequate mental representation of the field site. Further, a recent study of field trips has indicated an overall positive correlation between students’ mental representation of a field site and their field trip enjoyment and learning experience (Zhao & Klippel, 2019). This gap in mental representation may explain in part why high spatial ability students are more likely to show interest and succeed in place-based disciplines (Nazareth, Newcombe, Shipley, Velazquez, & Weisberg, 2019).

Our data suggest that the elevated perspective can enhance spatial learning in large-scale VEs, in particular for participants with low self-reported SOD. Given the potential of the elevated perspective to bridge the performance gap between low and high spatial ability learners in place-based learning, our research team has been working on integrating the elevated perspective with virtual field trips into geoscience classrooms of Penn State Commonwealth campuses. We have developed a virtual field trip that offers access to a field site from the elevated perspective using 360° images taken at 27 ft./8.2 m from the ground. Students’ feedback collected from the post-survey shows that compared to the normal ground level, students are in favor of the elevated perspective given its facilitatory role in perceiving the spatial layout of the field site (Zhao & Klippel, 2019).

## Limitations and future directions

Our results show that, when learning the virtual maze at ground level alone, low-SOD participants not only were less precise in their pointings but tended to make more systematic errors than high-SOD participants. Interestingly, the absolute pointing errors they made were not the same across different target locations. Their systematic errors were also not evenly occurred in the virtual maze. Tversky (1993) suggests that systematic errors are more easily made when an environment has irregular geographic features or a route has more turns or clutter. This implies the role of environmental complexity in spatial learning (Baumann & Mattingley, 2013; Carassa, Geminiani, Morganti, & Varotto, 2002). If the environment is too complex (e.g., a city), the information from different sources and in different forms may not be compatible, mental maps may take a long time to develop, and hence an external survey representation (e.g., a traditional map) is preferred. For a simpler environment (e.g., a stadium), in contrast, a rapid-forming representation that preserves coarse spatial relations from direct experiences may be perfectly adequate for the needs of individual concerned. Therefore, the amount of spatial information that learners need depends on environmental complexity and is not equivalent across an environment that contains distinct paths and irregular geographic features. The disparity in information needs seems to provide a goal-directed manner to reduce error. Instead of offering the elevated perspective at each location, we could help low spatial ability learners reduce systematic errors by increasing geographic scale specifically at parts of the environment that would possibly lead to distortions observed in the alternative model. Considering that low spatial ability learners are likely to be overwhelmed by excessive information (Cooper, 1998; Lee & Wong, 2014), reducing the amount of spatial information being visually accessible while preserving the key additional information for complex situations seems to be an efficient mechanism that allows them to reconcile the inconsistencies in spatial knowledge in the correct direction.

**Fig. 12:**
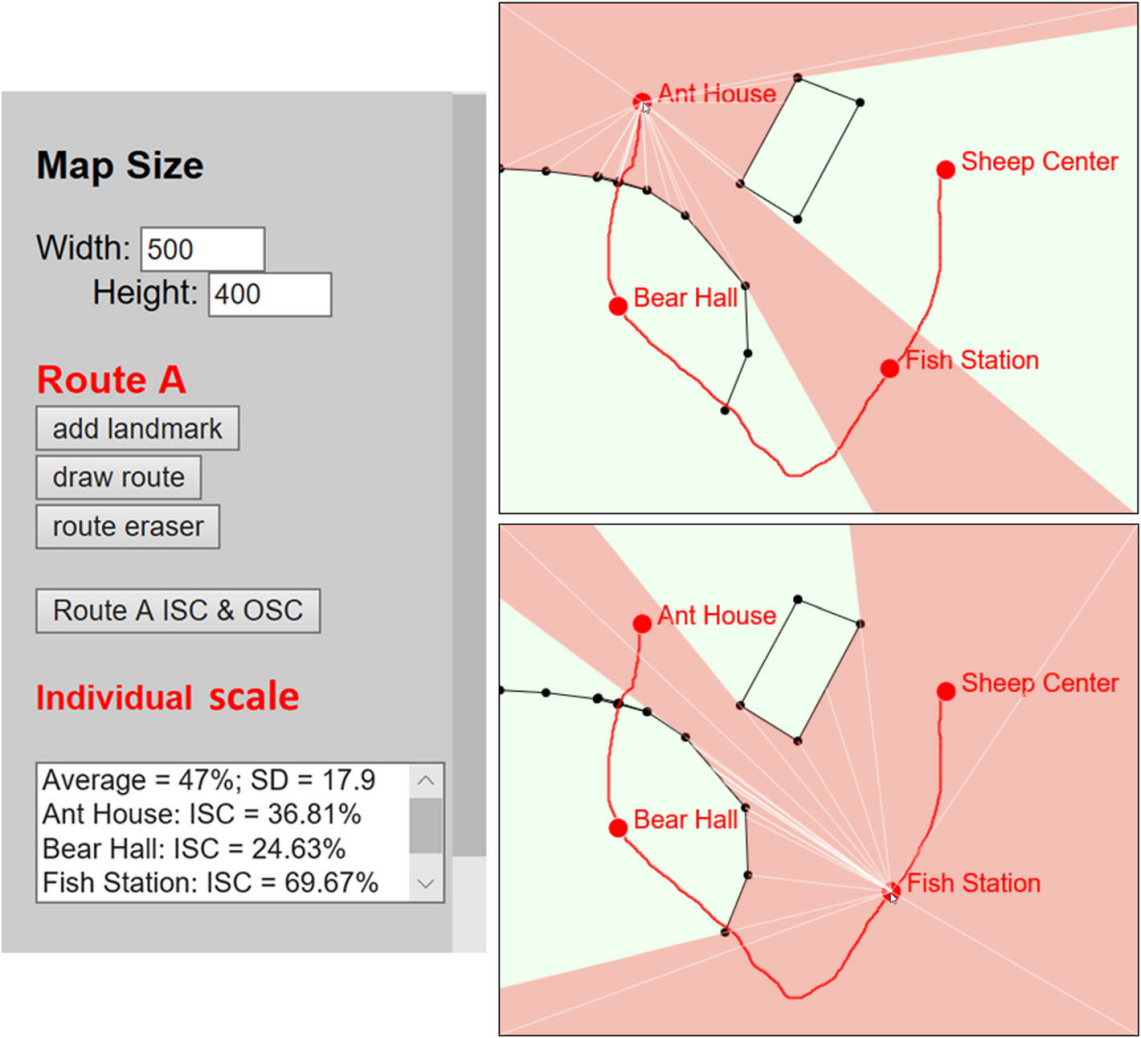
Prototype of interactive design of VEs. Left: user-defined parameters expressing the map size, landmark names and locations, and route layout. ISC represents geographic scale at individual locations. Right: Solid red dots denote viewpoints that are associated with self-defined locations. Top right: The spatial extent (semi-transparent red portion) visually accessible from the Ant House interacts with and is limited by visual barriers (line segments connected by black nodes). Bottom right: Viewpoint at the Fish station has a larger spatial extent being visually accessible, resulting in a larger geographic scale than the viewpoint at the Ant House

The findings reported in this paper are primarily dependent on experiences at ground versus elevated levels; confounding factors, such as the different angles of view brought by perspective change, cannot be ruled out. The study of Barra et al. (2012) identifies that, compared to the horizontal angle of view through experiences at ground level, the oblique angle of view from an elevated perspective can facilitate navigation and orientation in VEs. This may provide an extra advantage to spatial learning in the G + E or E groups. Alternatively, keeping participants in these groups staying on the ground while allowing them to see through the hedges along the route would help to eliminate the group difference in the angle of view (e.g., He et al., 2019; Piller, 2006). On the other hand, while geographic scale is a continuous concept, it was manipulated as two perspectives in the current study and was largely dependent on the number of landmarks that can be seen from a single viewpoint. Thus, we are not able to answer the question of to what exact extent geographic scale would affect spatial learning yet. Experiencing a space at specific scales above or below some critical values may have a drastically different learning outcome (Freksa & Barkowsky, 1996). Taken together, a more rigorous approach is needed to precisely manage the dynamic change of geographic scale. We are, therefore, in the process of developing a web-based application that enables interactive design of VEs for future experimental studies. The tool allows for creating a VE by drawing points of interest, routes, and visual barriers on a map with the geographic scale and azimuth of individual locations being updated and displayed in real time. Figure 12 shows the user interface of our prototype with a simplified design to illustrate how geographic scale varies with visual barriers.

The systematic error analysis and modeling used the pointing data to formalize and illustrate the construction of mental representations on the basis of non-random or systematic deviations from the correct representation of the virtual maze. In this study the unified alternative maze model was generated merely from observations of systematic error. Meilinger et al. (2018) suggest that pointings may not rely on one unified, but rather multiple representations of the environment. They further indicate that different sources of systematic error, such as forgetting segments or mixing up turns, provide reliable evidence for the underlying types of representations. Following Meilinger et al. (2018), a number of alternative models can be generated based on different sources of systematic error. We are in the process of developing an algorithm that allows for calculating and evaluating the fitting degree of these underlying models via systematic errors.

## Data Availability

The datasets used during the current study and the analysis code are available from the corresponding author on reasonable request.

## Abbreviations

E: Elevated
G + E: Ground + elevated
G: Ground
GLMM: Generalized linear mixed model
SBSOD: Santa Barbara Sense of Direction Scale
SOD: Sense-of-direction
TLX: Task Load Index
VE: Virtual environment

## Footnotes

1 In contrast to typical map representations, some mobile navigation applications (e.g., Google Maps) have the default option of auto-rotating the digital base map based on the user’s facing direction, enabling an egocentric map representation of the space.

2 360° images discretize locomotion. 360° imagery renders an environment partially as discrete viewpoints, forcing learners to be transited from one location to the next at essentially infinite velocity (i.e., teleportation).

3 Pointing error was estimated to be in whichever direction produced the least discrepancy, resulting in a maximum possible error of 180°.

4 https://www.oculus.com/go/

5 https://unity3d.com/

6 The purpose of repeated exposure and measures is to diminish the underlying novelty effect of virtual reality setup on participants’ performance.

7 Continuous rating scale is currently not supported by Google Forms.
